# Implementation of a hybrid healthcare model in rheumatic musculoskeletal diseases: 6-months results of the multicenter Digireuma study

**DOI:** 10.1186/s41927-023-00362-7

**Published:** 2023-09-25

**Authors:** D. Benavent, L. Fernández-Luque, M. Sanz-Jardón, I. Bilionis, M. Novella-Navarro, V. Navarro-Compán, P. L. González-Sanz, E. Calvo, L. Lojo, A. Balsa, Ch Plasencia-Rodríguez

**Affiliations:** 1 Department of Rheumatology, Hospital Universitario La Paz, IdiPaz, Madrid, Spain; 2 AdheraHealth Inc., Santa Cruz, CA USA; 3 Department of Rheumatology, Hospital Universitario Infanta Leonor, Madrid, Spain

**Keywords:** Mobile solution, Digital health, Rheumatoid arthritis, Spondyloarthritis

## Abstract

**Objectives:**

Rheumatic and musculoskeletal diseases (RMDs) require a tailored follow-up that can be enhanced by the implementation of innovative tools. The Digireuma study aimed to test the feasibility of a hybrid follow-up utilizing an electronic patient reported outcomes (ePROs)-based monitoring strategy in patients with RMDs.

**Methods:**

Adult patients with rheumatoid arthritis (RA) and spondyloarthritis (SpA) were recruited for a 6-month bicentric prospective follow-up consisting of face-to-face and digital assessments. Patients were asked to report disease-specific ePROs on a pre-established basis, and could also report flares, medication changes, and recent infections at any time. Four rheumatologists monitored these outcomes and contacted patients for interventions when deemed necessary. Results from face-to-face and digital assessments were described.

**Results:**

Of 56 recruited patients, 47 (84%) submitted any ePROs to the digital platform. Most patients with RA were female (74%, median age of 47 years), while 48% of patients with SpA were female (median age 40.4 years). A total of 3,800 platform visits were completed, with a median of 57 and 29 visits in patients with RA and SpA, respectively. Among 52 reported alerts, 47 (90%) needed contact, of which 36 (77%) were managed remotely. Adherence rates declined throughout the study, with around half of patients dropping out during the 6 months follow-up.

**Conclusion:**

The implementation of a hybrid follow-up in clinical practice is feasible. Digital health solutions can provide granular knowledge of disease evolution and enable more informed clinical decision making, leading to improved patient outcomes. Further research is needed to identify target patient populations and engagement strategies.

**Supplementary Information:**

The online version contains supplementary material available at 10.1186/s41927-023-00362-7.

## What is already known on this topic –

- While the interest of digital health technology has rapidly grown in recent years, the implementation of projects in rheumatology is still scarce.
- The use of patient-reported outcomes is critical in the follow-up of rheumatic diseases, and they can now be collected using electronic methods (ePROs).
- There is a need for evidence on the implementation of remote care strategies for monitoring via ePROs.

## What this study adds –

- The Digireuma project evaluated as feasible a hybrid -virtual and face-to-face- ePROs based monitoring strategy in patients with RA and SpA.
- Remote management of alerts generated through ePRO monitoring was successful for most cases, with only a small percentage requiring face-to-face interventions.
- Declining adherence rates were identified throughout the 6-month follow-up period.

## How this study might affect research, practice or policy –

- These findings suggest that a hybrid follow-up approach in clinical practice, using both face-to-face and digital solutions, is feasible and can provide more detailed understanding of disease evolution.
- Future research can identify patient populations that could benefit most from this type of follow-up and strategies that promote engagement across various sub-groups.

## Introduction

During the last few years, there has been an exponential growth in digital health technology, which aims to use communication applications to support the diagnosis, monitoring, treatment or prevention of disease for the general public [1]. Digital health has been applied in different forms in rheumatic and musculoskeletal diseases (RMDs), such as teleconsultation or mobile-based solutions [2]. The use of mobile applications provides an opportunity to change disease management through the collection of large amounts of data, since today most patients have ready access to smartphones. Although there have been some attempts to address the rheumatologic spectrum in general, monitoring patients with rheumatoid arthritis (RA) has thus far been the main area of interest [3]. Notwithstanding various successes, the development of monitoring strategies for other prevalent RMDs, such as spondyloarthritis (SpA), remains a priority for improving the management of rheumatologic consultations.

The use of patient-reported outcome measures (PROMs) is critical in RMDs, since they provide an assessment of patient disease activity and other relevant constructs, yielding actionable insights into a patient’s health in between clinical visits [4]. PROMs are particularly important in the evaluation of SpA, as physical examinations and laboratory tests have limitations in assessing this condition. In most patients with SpA, blood markers of acute phase reaction show no elevation. Therefore, self-reported measures of a patient's experience, symptoms, and functional status play a crucial role in providing a comprehensive understanding of the disease's impact on a patient's daily life. PROMs have historically been collected in paper forms during consultations. However, in recent years, novel collection methods have been tested. In this way, digital health initiatives have facilitated the collection of electronic patient-reported outcome measures (ePROs), which may be captured using patients’ mobile applications [5]. By utilizing a digital solution instead of the traditional paper questionnaires, clinicians can gain insights into patient symptoms that arise in between visits. This technology constitutes a major advance in the optimization of managed care for patients with RMDs.

The European Alliance of Associations for Rheumatology (EULAR) has recently published guidelines on the development and implementation of digital solutions and remote care in RMDs. EULAR points-to-consider (PtC) on the development, evaluation and implementation of mobile solutions aiding self-management of RMDs emphasize the involvement of patients and caregivers in creating mobile solutions, as well as transparency and accessibility [6]. Moreover, the EULAR PtC for the development, prioritization, and implementation of telehealth for individuals with RMD has also been published, highlighting areas where telehealth may improve healthcare quality and expand access to care [7]. The publication of these guidelines highlights the growing interest in the field of remote monitoring of RMDs. Of note, both publications were formulated as PtC (as opposed to recommendations), mainly due to the scarcity and weakness of existing evidence [8].

In this context, we identified the need for a remote care strategy that enables monitoring RMDs via ePROs. Thus, we sought to obtain evidence of the advantages of having a more granular view of a patient's clinical status between visits. By doing this, the Digireuma project aimed to assess the feasibility of a bicentric hybrid (virtual and face-to-face) utilizing an ePRO-based monitoring strategy in patients with RA and SpA. An interim analysis of one of the centers at three months was previously published [9]. The results showed that the use of a digital health solution was feasible, with a high level of patient satisfaction and the successful remote management of most alerts generated. The current manuscript presents the results of the final analysis at the end of follow-up (6 months), including data from both hospitals.

## Methods

Before the start of the prospective study, a mixed-care model (MAM) was designed for monitoring patients with RMDs treated with biologic or targeted synthetic disease-modifying anti-rheumatic drugs (b/tsDMARDs). This strategy combines traditional in-person appointments with self-monitoring at home using a digital solution. The MAM was adapted for a clinical practice protocol; details are described elsewhere [9]. The follow-up process involved the completion of ePROs through which patients provided with regular updates on their health status. A dedicated rheumatologist regularly reviewed incidents recorded in the system through a web interface, minimum in a bi-weekly basis. If necessary, patients were contacted by phone to address any reported incidents, which could be resolved either remotely- leading to minor treatment adjustments- or through an in-person visit with the on-site clinician. In parallel, patients also received face-to-face visits according to our department’s standard procedures, typically scheduled at approximately three-month intervals. The study protocol was registered prior to the start of recruitment (ISRCTN11896540). The aim of this protocol is to establish a systematic telematic monitoring system in routine clinical practice, enabling clinical follow-up beyond scheduled appointments.

### Patients

Patients aged 18 years and older with RA or SpA as diagnosed by the treating rheumatologist, and who were treated with b/tsDMARDs were included consecutively. Inclusion criteria were age > 18 years and ability to use a mobile phone. Patients were recruited at two hospitals in Madrid, Spain: Hospital Universitario La Paz (HULP) and Hospital Universitario Infanta Leonor (HUIL). The recruitment period at HULP was June-July 2021, while at HUIL it ran from December 2021-January 2022.

### Mobile solution

An existing digital health solution (Adhera® Precision Digital Companion Platform™) was adapted for use with RA and SpA patients. Patients using this MAM were provided access to a digital application called the Adhera® Rheumatology Digital Program, which included various elements to support their self-managed care. The process of adapting this digital solution encompassed interdisciplinary work involving rheumatologists, a general physician, psychologists, and digital health specialists. Patients downloaded Adhera’s app onto their mobile devices, where they could access content and complete ePROs at home. In addition, the mobile application provided patients with educational materials and capabilities to support self-management skills. A set of customized motivational messages were delivered as part of the solution. The educational material, support for self-management, and motivational messages included in the digital health solution were prepared at the start of the implementation and were not updated during the study period.

### Study design

Digireuma was a 6-month prospective study. The enrolled patients had a hybrid follow-up consisting of face-to-face (basal, 3 and 6 months) and digital (self-reported at home, both on demand and at established timepoints) assessments. For all patients, baseline data were defined as the time point when they downloaded the application. The follow-up period was defined from baseline until six months later.

### Data sources

Patients were asked to report disease-specific ePROs on a pre-established basis within the Adhera® Rheumatology Digital Program. In addition to flares, any incidences with medication and/or recent infections were assessed. Four rheumatologists (two at each center) monitored these outcomes, and patients were contacted for online or face-to-face interventions when deemed necessary by clinicians. PROs and clinical activity scores were also performed during face-to-face visits. Additionally, sociodemographic, and clinical information from electronic health records (EHRs) were compiled from the two hospitals.

### Clinical outcomes

ePROs for patients with RA included the following: patient global assessment (PtGA) of disease activity, self-reported tender joint count (s-TJC), self-reported swollen joint count (s-SJC), Health Assessment Questionnaire (HAQ) and pain visual analogue scale (VAS). Self-report joint counts have proven to effectively record involvement of specific joints in RA and PsA [10]. In face-to-face visits, joint counts were performed by the rheumatologist, and a disease activity score (DAS28) was added to the previous outcomes. In the case of patients with SpA, the following PROs were monitored both digitally and face-to-face: PtGA, Bath Ankylosing Spondylitis Disease Activity Index (BASDAI), s-TJC, s-SJC; while the ASAS Health Index (ASAS-HI) was only monitored digitally, since it was not implemented in clinical practice. All ePROs were delivered every two weeks, thereby avoiding two questionnaires on the same day (Supplementary Table 1). The information on all the questionnaires was extracted from the website https://oml.eular.org/.

### Additional collected data

In addition, patient self-reported incidences – including flares, incidences with the medication and recent infections – were available on-demand and could be reported at any time. The total number of such incidences, which appeared as alerts in the clinician interface, were assessed. Platform visits were defined as each instance a user logged into the solution. Interaction with the solution was defined as any change of content on the user’s screen, including access to the questionnaires, the motivational messages or to the educational content. The total number of platform visits, interactions and completion of ePROs were used to assess engagement with the solution.

On-site PROs were completed at baseline and at the 6-month face-to-face visit. A routine clinical visit at 3 months without completing specific questionnaires was made.

### Statistical analysis

Baseline data regarding demographic and clinical outcomes were analyzed. Follow-up analyses of PROs were performed at 6 months for face-to-face and at 3 and 6 months for digital assessments, using temporal windows of ± 15 days around these dates. Engagement with the digital solution was considered using the same time windows. Frequency tables were used to describe categorical variables, which were expressed by n (%). Continuous variables are presented by means of summary tables that include median and interquartile ranges (IQRs).

### Ethics

The study was approved by the Ethics committee of the participating sites. Informed consent was obtained from all subjects prior to their participation. All methods were performed in accordance with the relevant guidelines and regulations, including the Declaration of Helsinki.

## Results

### Characteristics of participants

**Fig. 1:**
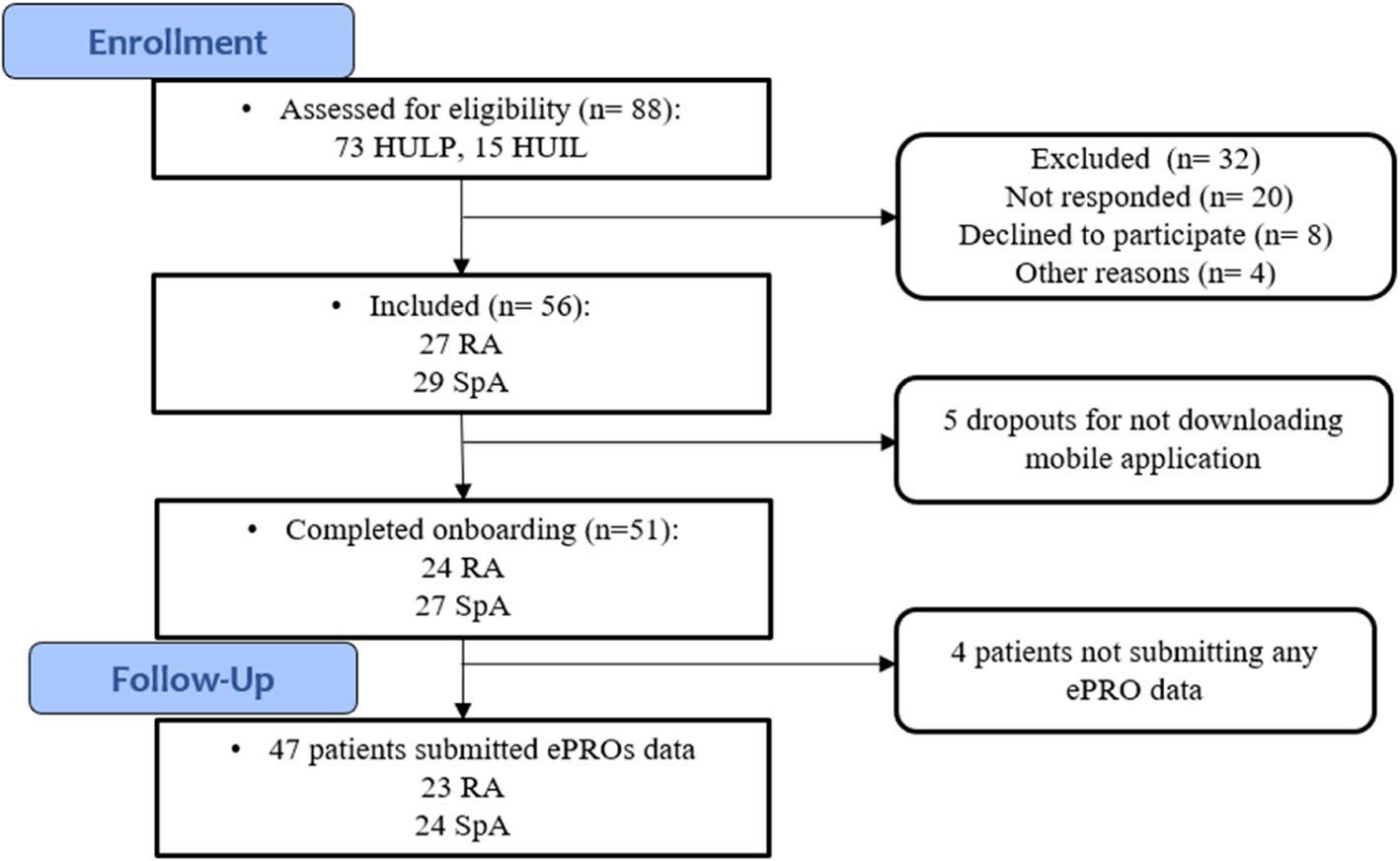
Flowchart of the prospective study. SpA: spondyloarthritis; RA: rheumatoid arthritis. HULP: Hospital Universitario La Paz. HUIL: Hospital Universitario Infanta Leonor. ePRO: electronic patient reported outcome

A total of 88 patients were contacted, of which 32 were excluded for different reasons (Fig. 1). Overall, 56 adults were recruited (RA = 27; SpA = 29). Fifty-one patients (RA = 24; SpA = 27) completed the onboarding process and actively used the Adhera Rheumatology Digital Program. Of the 51 active users, 47 (RA = 23; SpA = 24) submitted some data (either in ePROs or incidences) to the Digireuma study.

Concerning the characteristics of patients with RA, 20/27 (74.1%) were female, with an overall median age (IQR) of 47.0 (13.2) years. Regarding patients with SpA, 14/29 (48.3%) were female and the medium age was 40.4 (6) years. Of the 29 participants with SpA, two presented purely axial SpA and two peripheral SpA. The predominant pattern was seen in 25 patients who presented with both axial and peripheral symptoms. Baseline clinical and demographic data are displayed in Table 1.

**Table 1:** Clinical characteristics stratified by disease at digital solution baseline

	**RA patients (** ***n*** ** = 27)**	**SpA patients (** ***n*** ** = 29)**
**Demographic and clinical features**		
** Sex (female)**	20 (74.1)	14 (48.3)
** Age (years)**	47.0 (13.2)	40.4 (6)
** Time since biologic onset (years)**	7.9 (4.8)	5.8 (5.2)
** Smoking habit (ever smoker)**	13 (50.0)	11 (42.3)
** RF positive**	19 (70.4)	-
** ACPA positive**	21 (77.8)	-
HLA*B27 positive	-	14 (58.3)
**Concomitant treatment**		
** csDMARDs**	23 (85.2)	12 (44.4)
** Prednisone**	11 (40.7)	0

*ACPA* anti-citrullinated peptide antibodies, *RF* rheumatoid factor, *csDMARDs* conventional synthetic disease-modifying antirheumatic drugs, *SpA* spondyloarthritis, *RA* rheumatoid arthritis. Results are expressed in median (IQR) and n (%)

### Outcomes at baseline and follow-up

Outcomes reported at baseline and follow-up are shown in Table 2. Patients with RA presented good disease control throughout the study, both measured at face-to-face consultations and remotely via ePROs. Baseline DAS-28 at face-to-face consultations was 2.6 (2), with TJC: 1 (3.25) and SJC: 0.5 (2.5). Digital-solution joint counts (i.e. performed by the patient at home) at baseline, s-TJC and s-SJC, were numerically higher compared to face-to-face assessments: 1 (1.75) and 3 (3.55), respectively. Nonetheless, mean DAS-28 was maintained in remission status during the different timepoints through follow-up.

**Table 2:** Outcomes in clinical face-to-face and digital visits at baseline and follow-up

	**Face-to-face visits**		**Digital visits**		
	**Baseline**	**6 months**	**Baseline**	**3 months**	**6 months**
**Rheumatoid Arthritis ** ***n*** ** = 23**					
DAS28	2.6 (2)	1.6 (1.8)	-	-	-
TJC	1 (3.25)	0 (2)	1 (2)	1 (0.5)	1 (0.8)
SJC	0.5 (2.5)	0 (2)	1 (1.5)	1 (0)	1 (0.8)
HAQ	0.13 (0.9)	0.13 (0.63)	0.2 (0.5)	0.4 (0.4)	0.25(0. 4)
PtGA	2 (4)	1.5 (3.5)	2 (1.7)	2 (2.5)	2 (0.8)
VAS pain	4 (3)	1.35 (2.25)	3 (5)	1.5(1.2)	2 (1)
**Spondyloarthritis ** ***n*** ** = 24**					
BASDAI	2.1 (3)	2.2 (3.6)	3.3 (5.3)	1.4 (4.2)	2.2(0.6)
TJC	0 (1)	0 (2)	1 (1.75)	3.5(2.5)	2.5(0.6)
SJC	0 (1)	0 (0.25)	3 (3.6)	1.5(1.75)	5 (4)
PtGA	2 (3)	2 (4)	1 (1.5)	4.5 (3)	2.5(1.8)
VAS pain	2.5 (3)	2 (3.25)	-	-	-

*DAS28* Disease Activity Score-28, *BASDAI* Bath Ankylosing Spondylitis Disease Activity Index, *TJC* tender joint count, *SJC* swollen joint count, *HAQ* health assessment questionnaire, *PtGA* patient global assessment, *VAS* Visual analogue scale. Results are expressed in median (IQR)

Patients with SpA presented a median baseline BASDAI of 2.1 (3) at face-to-face consultations, while median reported BASDAI in the digital solution measured 3.3 (5.5). In addition, PtGA at consultation was 2 (1.7), while it was 1 (1.5) in the solution. Nonetheless, results for both outcomes were more similar to those recorded at 6 months. In contrast, both TJC and SJC yielded higher numeric results in the solution compared to face-to-face assessments at baseline or follow-up.

### Management of notifications

Concerning electronic alerts, of 52 notifications, 47 were classified as warranting contact, while in 5 cases it was decided that an assessment could wait until the already programmed consultation, due to the time proximity. Of all the alerts, 45 were flares (31 RA, 14 SpA) while 4 involved medication problems (3 causes were not registered). Of the 47 cases requiring contact, 36 (77%) were managed remotely, 9 (19%) involved a face-to-face intervention and in 2 (4%) cases it was not possible to reach the patients prior to their consultation.

### Use of the mobile solution

**Fig. 2:**
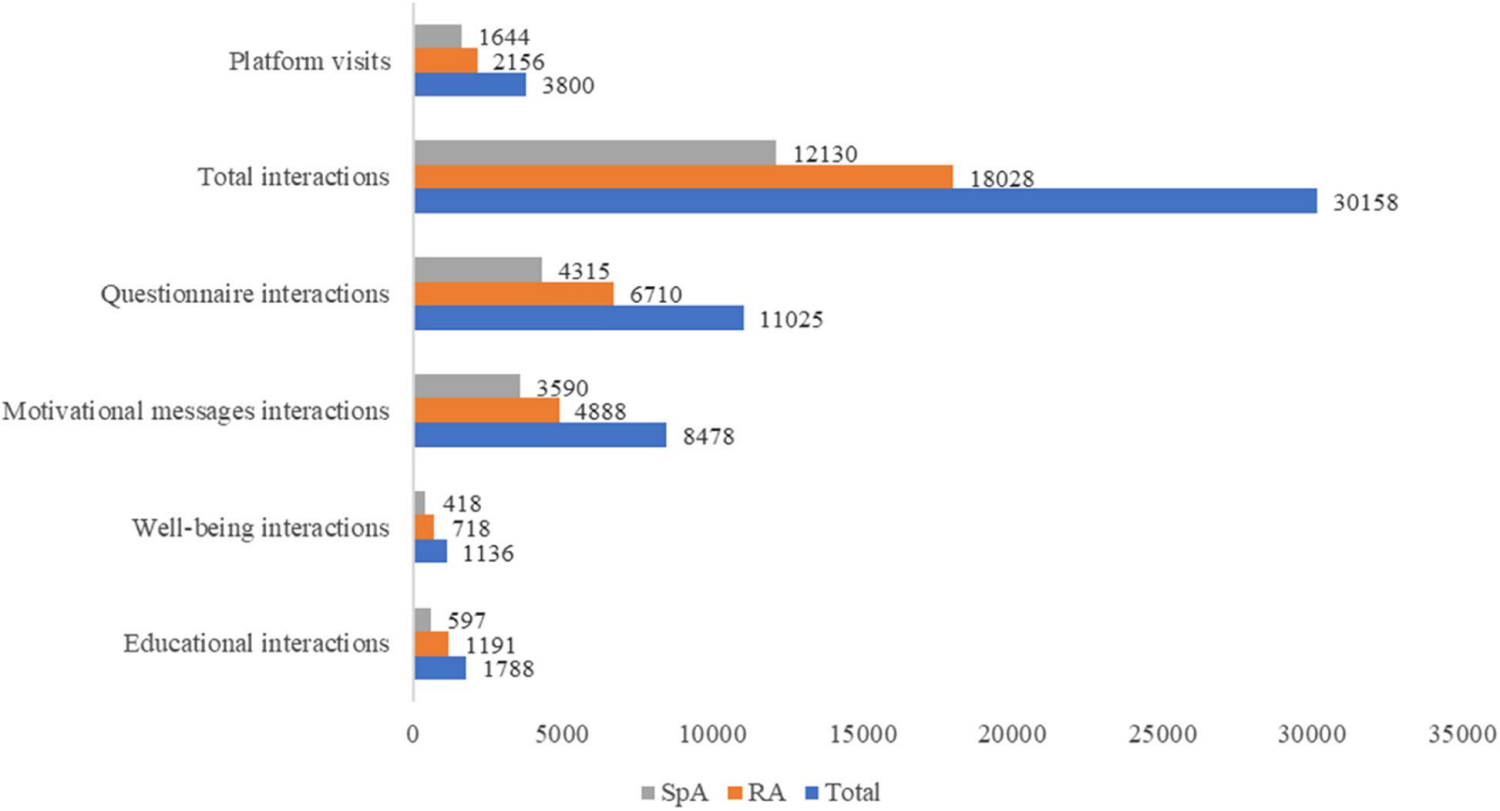
Total number of patient interactions with the digital solution. SpA: spondyloarthritis; RA: rheumatoid arthritis

Metrics on use of the digital program during the study period are reported in Fig. 2 (complete data in Supplementary Table 2). The total number of visits was 3,800; 2,156 by patients with RA (57 [113.5] visits per patient) and 1,644 by those with SpA (29 [45] visits per patient). In general, patients with RA made more frequent use of the mobile solution compared to those with SpA. Thus, a median (IQR) of 814 (740) interactions were completed by patients with RA, and a median of 245 (655) with SpA. Of the different types of interactions, “questionnaires” ranked highest in both groups, with 193 (345) entries in patients with RA and 43 (213) in those with SpA.

Concerning ePROs completion during follow-up, Supplementary Table 3 shows onboarded patient engagement. The most frequently completed ePRO for patients with RA was VAS pain, with a completion rate of 35.1%; for those with SpA, PtGA, with a completion rate of 22.7%. At three months 26 patients (55%)- 15 with RA and 11 with SpA- continued submitting data periodically, while at six months 22 patients (47%)- 13 with RA and 9 with SpA- had submitted some data during the previous 15 days.

## Discussion

The Digireuma study tested a digital health solution to track ePROs and monitor the status of patients with RA and SpA. Of the 56 included patients, 47 submitted monitoring data throughout the 6-month follow-up period. While there were similarities in the median results between face-to-face and digital assessments for most outcomes, this did not hold true universally across all outcomes and timepoints. Notably, differences were observed in baseline BASDAI scores and in TJC and SJC assessments in SpA patients. Thus, the values of ePROs and PROs in clinical practice were comparable, indicating the utility of ePROs as a very useful tool for patient assessment and monitoring, one that can not only enhance the accuracy and completeness of patient-reported data, but also enable more informed clinical decision making and improved patient outcomes. On the other hand, joint counts assessments for both diseases were higher in the mobile solution compared to the face-to-face assessments. Of the 47 alerts that resulted in contact, 36 were managed remotely and 9 required a face-to-face intervention. Participants completed a total of 3,800 platform visits, with a median of 57 and 29 visits per patient with RA and SpA, respectively. The most frequently completed ePROs were VAS pain and PtGA. Declining adherence rates were evident throughout the study, with more than half of patients dropping out during follow-up at 6 months. As described below, other studies have discussed the various factors that can affect adherence to these types of digital solutions, [11, 12]. However, inter-study comparisons are difficult due to the uncontrolled nature of most such studies (e.g., lack of common implementation strategies). Our data suggests that the implementation of a hybrid follow-up in clinical practice is possible, and can provide more granular knowledge of disease evolution. Further research might help identify not only which patient populations could most benefit from this type of follow-up, but also those strategies that foster engagement across various sub-groups.

The significant advantages of smartphone data collection for both research and clinical care have recently been described, including the use of ePROs to guide clinicians and enable patients to better report their symptoms [13]. Nonetheless, rheumatologists and researchers in RMDs have emphasized the need for more carefully calibrated apps that translate data into quantitative clinical outcome values [14, 15]. A systematic review from 2020 identified only one clinical trial that included a smartphone app for patient monitoring [16], which was a mixed-methods pilot study that aimed to facilitate the self-management of RA. This trial involved 21 participants who required some form of intervention and 15 controls [17]. More recently, increasingly robust evidence on the reliability and effectiveness of mobile solutions has been published. Uhrenholt et al. developed a randomized, within-participants, crossover, agreement trial to demonstrate equivalence among different PROMs for two device types- including a smartphone app- among patients with inflammatory arthritis [5]. The results of this study showed that the two device types yielded equivalent results for all tested PROMs, with the exception of BASDAI (although even here the difference was within minimally significant bounds). In our study, most outcomes had comparable median results in face-to-face versus digital solution assessments. However, TJC and SJC showed consistently worse results in the digital assessments, which highlights the relevance of patient education for optimal self-assessment. These findings underscore the importance of training patients in correct self-examination, which can only lead to greater knowledge of their disease. Concerning effectiveness, Seppen et al. performed a randomized, non-inferiority clinical trial comparing app-supported care to standard care. The primary outcomes were non-inferiority in terms of changes in DAS28 and the number of consultations with rheumatologists. The results showed that the app was non-inferior to standard care in terms of disease activity and, moreover, led to a 38% reduction in rheumatologist consultations [18]. More recently, a pivotal multicenter, open-label randomized clinical trial conducted across 22 tertiary hospitals in China further substantiated the value of digital health applications in RA management. The trial compared a smart system of disease management to conventional care and found a significant increase in disease control rate at the 6-month mark in the system of disease management group [19]. This echoes our findings of similar performance between digital and face-to-face assessments over time, underscoring the potential of digital health solutions in enhancing disease management. Although our study was not designed to assess the effectiveness of the digital solution, given that three out of four alerts were managed remotely, it is reasonable to believe that the necessity of such consultations may have been reduced.

Technological advancements are offering unprecedented avenues for refining disease activity monitoring. Recent studies demonstrate the potential of smartphone-based joint count recognition, employing convolutional neural networks for the detection and monitoring of joint swelling in RA patients [20]. Similarly, self-sampling methodologies for capillary blood collection have shown encouraging results, enabling remote monitoring of inflammation markers and autoantibodies in RA [21]. Additionally, another innovative approach has been the use of thermography, a fast and non-invasive imaging technique, coupled with machine learning to automatically assess joint inflammation in RA patients. The Thermographic Joint Inflammation Score (ThermoJIS), derived from this method, showed a moderate correlation with ultrasound scores and was able to detect active synovitis even in patients in clinical remission [22]. These approaches offer a unique advantage: they can be performed by patients at their convenience, reducing the burden of travel and frequent clinical visits, while providing valuable data for disease management. Furthermore, these technologies provide opportunities for patients to engage actively with their disease management and develop a greater understanding of their condition. These advancements in digital health, along with the digital solutions explored in our study, are crucial components of a future, patient-centered care model. Subsequent studies should look into the acceptability of new telemedicine services and its impact in engagement; these can include video conferencing and other types of patient-generated data such as wearable devices and patient captured photos.

Engagement with mobile health solutions requires substantial effort on the parts of both researchers and participants [23]. In this sense, attrition might be a significant threat to the validity and generalizability of research findings, as it can lead to a biased sample. In previous studies that have used mobile apps to collect ePROs, initial adherence to reporting has been high, but has often declined over time. For example, one study found that adherence dropped from 88 to 62% over a 6-month period [24], while another reported that adherence rates fell from over 90% in the first week to less than 50% by the fourth week [12]. Interestingly, a more recent study reported that out of 220 consecutive patients with either RA or SpA (including psoriatic arthritis and axial SpA) invited to telemonitor their disease activity, 64% dropped out, with a median drop-out time of 17 weeks [11]. Our data is consistent with this study, showing that at three months half of patients continued to submit data periodically, while at six months, only one third continued to do so. These data reveal that maintaining patient engagement throughout follow-up is one of the greatest challenges in digital health monitoring. From our experience, we have drawn two conclusions: the importance of involving patients in these technological advances in order to better identify their needs and how the creation of personalized reinforcements supported by artificial intelligence models can enhance engagement. As part of future research, we will study participant perceptions on aiming at identifying among participants features and virtual care services that they would like to add or delete. Our forthcoming research aims to investigate the participant characteristics that are associated with higher adherence to digital programs. Additionally, we are exploring the correlation between engagement and the interaction with various elements of a mobile application. Preliminary findings indicate that the use of ePROs is connected to increased engagement, potentially due to the importance of monitoring in the hybrid care model. Recent qualitative research on ePROs adherence in rheumatic conditions identifies key factors such as the frequency of ePROs, result discussions, insights derived from ePROs, and the overall user experience [25]. Moreover, studies have demonstrated a statistical link between patient characteristics, such as gender and health status, and higher dropouts in terms of engagement [26]. Our future research will further investigate these factors, including the relationship between different elements of the solution and adherence to the mobile solution. Besides, future studies should focus on additional actions that could be undertaken to mitigate the risk of dropout, such as tracking attrition carefully, or focusing on recruiting those patients who might benefit the most from the solution.

The Digireuma study is a promising start towards integrating remote monitoring into rheumatology care, although some limitations must be acknowledged. First, as the study was conducted at two university hospitals involving patients with RA and SpA with a modest sample size, the generalizability of the results for other settings and musculoskeletal diseases remains limited. Besides, only patients with b/tsDMARDs were included, which may hinder the extrapolation to the results. It is not clear whether the use of this solution will lead to better clinical outcomes, as the study design does not allow for determining the effectiveness of this particular intervention. In addition, we did not test reliability due to the fact that assessments could only be carried out at varying time points in patients' evolution, which does not allow for any calculation of test–retest reliability. Nonetheless, the description of outcomes at different time points gives an overview of the patients’ status- and provides some indications on how face-to-face and digital assessments may influence their results. At baseline evaluation, we identified a notable disparity between TJC and SJC assessments in patients with SpA, which underscores the need for refining our approach to patient self-assessment by improving patient education, thus forming a pivotal area of focus for the successive phases of our project. Patient engagement with the mobile solution was a challenge throughout the project, in which we should improve patient education for self-examination. with only few of them completing most of the assessments, which was lower than expected. However, as mentioned above, this is consistent with the latest studies on digital health in rheumatology. The investigation of features related to non-adherence and the definition of the most suitable patient profile for mobile app use should be among the focuses of any future studies.

Mobile applications offer the potential to improve rheumatology care by collecting large amounts of data using ePROs. In this study, we show how ePROs can be used to monitor disease activity, flares, and medication issues in patients with RA and SpA, with three out of four alerts being managed remotely. However, around two-thirds of the patients were non-adherent to the solution at six months. The gathering of high frequency data in real-time for monitoring clinical outcomes could form the cornerstone for the future rheumatology care, although it would require the participation of both healthcare professionals and patients [27]. Our findings suggest that, while ePROs can be effective for tracking the activity and treatment of RA and SpA, the involvement of patients and proper training on self-assessment is crucial for successful evaluation of their health status and successful implementation of this mobile health technology in clinical practice.

### Supplementary Information

**Additional file 1: Supplementary Table 1.** Frequency of assessment of ePROs. **Supplementary Table 2.** Metrics of mobile solution use concerning the median use per patient of the digital solution in the 6-month follow-up. **Supplementary Table 3.** Onboarded patient engagement in regard to ePROs.

## Data Availability

The data underlying this article will be shared on request to the authors.
