# Supplementary material for: Implementation of a hybrid healthcare model in rheumatic musculoskeletal diseases: 6-months results of the multicenter Digireuma study
Source: BMC Rheumatol. 2023 Sep 25;7:32. doi: 10.1186/s41927-023-00362-7 (PMC10518964; doi:10.1186/s41927-023-00362-7)
Supplement: Supplementary file 1 — Additional file 1: Supplementary Table 1. Frequency of assessment of ePROs. Supplementary Table 2. Metrics of mobile solution use concerning the median use per patient of the digital solution in the 6-month follow-up. Supplementary Table 3. Onboarded patient engagement in regard to ePROs. [file 41927_2023_362_MOESM1_ESM.docx]

**Supplementary Table 1.** Frequency of assessment of ePROs

| **Rheumatoid Arthritis** | Spondyloarthritis |
| --- | --- |
| s-TJC: Every odd week on Mondays | s-TJC: Every odd week on Mondays |
| s-SJC: Every odd week on Mondays | s-SJC: Every odd week on Mondays |
| PtGA: Every odd week on Mondays | PtGA: Every odd week on Mondays |
| VAS pain: Every odd week on Tuesdays | VAS pain: Every odd week on Tuesdays |
| HAQ: Every even week on Mondays | BASDAI: Every odd week on Tuesdays |
| Physical activity (30 mins/ 3 times a week): Weekly on Sundays | ASAS HI: Every even week on Mondays |
| Past joint symptoms in last week: Monthly on Saturdays | Physical activity **(**30 min**s/** 3 times a week**)**: Weekly on Sundays |
| EMA anxiety: Weekly on Thursday and Thursday | Past joint symptoms in last week: Monthly on Saturdays |
| EMA depression: Weekly on Thursday and Thursday | EMA anxiety: Weekly on Thursday and Thursday |
| EMA fatigue: Weekly on Thursday and Thursday | EMA depression: Weekly on Thursday and Thursday |
| RA Flare: Always available | EMA fatigue: Weekly on Thursday and Thursday |
| Last 2 weeks treatment arthritis: Every odd week on Sundays | SpA Flare: Always available |
|  | Last 2 weeks treatment arthritis: Every odd week on Sundays |

SpA: spondyloarthritis; RA: rheumatoid arthritis; s-TJC: self-assessed Tender Joint Count; s-SJC: self-assessed Swollen Joint Count; PtGA: Patient Global Assessment of disease activity; VAS: Visual Analogue Scale; HAQ: Health Assessment Questionnaire; BASDAI: Bath Ankylosing Spondylitis Disease Activity Index; ASAS HI: Assessment of SpondyloArthritis International Society Health Index; EMA: Ecological Momentary Assesment

**Supplementary Table 2**. Metrics of mobile solution use concerning the median use per patient of the digital solution in the 6-month follow-up.

|  | Total | RA | SpA |
| --- | --- | --- | --- |
| Platform visits | 46.5 [22.75- 103] | 57 [39-152.5] | 29 [16 -61] |
| Total interactions | 390[144.5-939.8] | 814[373.5-1113] | 245[61-716] |
| Educational interactions | 10.5 [3 - 40.3] | 33 [14-90] | 5 [2-15] |
| Well-being interactions | 12[2-25] | 16 [4-41.5] | 5[0-20] |
| Motivational interactions | 91.5 [ 28.5 - 243.5] | 204 [79.5-288] | 40 [ 6 - 234] |
| Questionnaire interactions | 130.5 [ 33.5 -330] | 193 [121-466.5] | 43 [20 - 233] |

Results are expressed in median per patient [Q1-Q3]; RA: rheumatoid arthritis; SpA: spondyloarthritis.

**Supplementary Table 3.** Onboarded patient engagement in regard to ePROs

| Rheumatoid Arthritis (n= 27) | | | |  |
| --- | --- | --- | --- | --- |
|  | PtGA | VAS pain | HAQ | RA symptoms |
| ePROs completed (adherence) | 3.74 (31.2%) | 4.21 (35.1%) | 3.56 (29.7%) | 1.82 (30.4%) |
| Median | 3 [4.5] | 3 [5.5] | 3 [5] | 2 [3] |
| **Spondyloarthritis (n=29)** | | | | |
|  | PtGA | VAS pain | BASDAI | ASAS-HI |
| ePROs completed | 2.72 (22.7%) | 2.51 (21.0%) | 2.03 (17.0%) | 2.62 (21.8%) |
| Median | 2 [4] | 1 [3] | 1 [2] | 2 [3] |

Follow-up period was 6 months. Results are expressed in median (IQR) and n (%). PtGA: Patient Global Assessment of disease activity; VAS: Visual Analogue Scale; HAQ: Health Assessment Questionnaire; BASDAI: Bath Ankylosing Spondylitis Disease Activity Index; ASAS HI: Assessment of SpondyloArthritis International Society Health Index; RA: rheumatoid arthritis
